# Physiological Stress Mediates the Honesty of Social Signals

**DOI:** 10.1371/journal.pone.0004983

**Published:** 2009-03-25

**Authors:** Gary R. Bortolotti, Francois Mougeot, Jesus Martinez-Padilla, Lucy M. I. Webster, Stuart B. Piertney

**Affiliations:** 1 Department of Biology, University of Saskatchewan, Saskatoon, Canada; 2 Estación Experimental de Zonas Áridas, CSIC, Almeria, Spain; 3 IREC (CSIC-UCLM-JCCM), Ciudad Real, Spain; 4 School of Biological Sciences, University of Aberdeen, Aberdeen, United Kingdom; University of Oxford, United Kingdom

## Abstract

**Background:**

Extravagant ornaments used as social signals evolved to advertise their bearers' quality. The Immunocompetence Handicap Hypothesis proposes that testosterone-dependent ornaments reliably signal health and parasite resistance; however, empirical studies have shown mixed support. Alternatively, immune function and parasite resistance may be indirectly or directly related to glucocorticoid stress hormones. We propose that an understanding of the interplay between the individual and its environment, particularly how they cope with stressors, is crucial for understanding the honesty of social signals.

**Methodology/Principal Findings:**

We analyzed corticosterone deposited in growing feathers as an integrated measure of hypothalamic-pituitary-adrenal activity in a wild territorial bird, the red grouse *Lagopus lagopus scoticus*. We manipulated two key, interrelated components, parasites and testosterone, which influence both ornamentation and fitness. Birds were initially purged of parasites, and later challenged with parasites or not, while at the same time being given testosterone or control implants, using a factorial experimental design. At the treatment level, testosterone enhanced ornamentation, while parasites reduced it, but only in males not implanted with testosterone. Among individuals, the degree to which both parasites and testosterone had an effect was strongly dependent on the amount of corticosterone in the feather grown during the experiment. The more stressors birds had experienced (i.e., higher corticosterone), the more parasites developed, and the less testosterone enhanced ornamentation.

**Conclusions/Significance:**

With this unique focus on the individual, and a novel, integrative, measure of response to stressors, we show that ornamentation is ultimately a product of the cumulative physiological response to environmental challenges. These findings lead toward a more realistic concept of honesty in signaling as well as a broader discussion of the concept of stress.

## Introduction

According to sexual selection theory, extravagant ornaments evolved to advertise their bearers' quality [1], such as heritable parasite resistance [2]. The Handicap Principle proposes that the honesty of social signals is ensured by the cost paid to produce or maintain their expression, thus avoiding “cheating” and reinforcing the evolutionary stability of the signaling system [3], [4]. Evolutionary biologists have been paying increasing attention to the role played by neuroendocrine hormones in the development of ornaments as social signals. Not only are they key endocrine influences on social behavior, but the interactions between the neuroendocrine system, the immune system, morphology and behavior could explain the honest development of ornamental traits [5]. Particular attention has been paid to the role played by testosterone [6]. Folstad and Karter (1992) proposed the Immunocompetence Handicap Hypothesis (IHH) whereby testosterone-dependent ornaments reliably signal health and parasite resistance because testosterone enhances ornamentation but impairs immune function, so only individuals of high genetic quality can endure the cost of displaying large ornaments. However, the evidence to date that testosterone is directly involved in immunosuppression is equivocal and its link to ornamentation has mixed support [7].

There are several explanations for the lack of consistent results with regards to the importance of testosterone to social signals, some of which question the mechanism itself. The impact of testosterone on immune function and parasite resistance might involve an indirect physiological pathway [8]–[10], where other agents, such as stress hormones (i.e. glucocorticoids), might play a more direct role [8], [11]. Alternatively, testosterone-dependent ornament expression may be contingent on some component of the environment, here used in the broad sense of the many abiotic and biotic components of an animal's life. Such unaccounted for variation, within or among studies, could thus be in part responsible for the mixed support for the IHH. We propose that an understanding of the interplay between the individual and its environment is crucial for interpreting the honesty of social signals.

The natural world is rife with environmental perturbations or stressors, or noxious stimuli [12], that challenge homeostasis (e.g. inclement weather, predators, parasites, social conflicts). How an individual copes with stressors is likely a major determinant of its overall well-being and health [11], [13]. For vertebrates, a major adaptation is the hypothalamic-pituitary-adrenal (HPA) axis, which releases glucocorticoid hormones in response to stressors, allowing individuals to recover from these perturbations in the best possible condition [14]. Many bad weather events and predator attacks can be considered as punctuated stressors. Other stressors like competition for territories or mates, or parasite infections, typically have a prolonged and seasonal component (see below) and thus have the real potential to induce a state of chronic stress. In such cases, glucocorticoids can have serious negative physiological consequences, perhaps the best known and most relevant of which here is immunosuppression [11], [15], [16]. These negative consequences may very well be the cost paid to maintain the honesty of the social signal [3], and hence exert a direct influence on the development of the ornament. However, given trade-offs for energy and resources [17], the response to stressors might impinge on the body's ability to produce the necessary biochemical components (e.g., testosterone) or function in combating parasites (e.g., immunocompetence). Therefore, the degree of exposure and response to stressors may be crucial as contextual information to understand the efficacy of, and to properly test for, other more direct mechanisms influencing the development of ornaments.

A major problem to date has been an ecologically meaningful measure of the response to stressors and how often they occur in nature. Blood levels of glucocorticoids, by virtue of their instantaneous sample and logistical limitations of capturing birds, cannot encapsulate effects of a temporally dynamic environment. With the aim of solving this limitation, it was recently discovered that the main avian glucocorticoid, corticosterone, can be measured reliably in feathers [18]. The amount of this hormone deposited in a growing feather provides an integrated measure of HPA activity; in effect, the sum over time of basal corticosterone variation and of the corticosterone released in response to stressors during a known time period (the growth of the feather). We thus asked the question whether considering individual variation in corticosterone might elucidate how other purported mechanisms interact and impact on sexual ornamentation. To answer this, we used a factorial experimental design and manipulated two key, interrelated components, parasites and testosterone, which influence both fitness and ornamentation, while measuring feather corticosterone, as an integrated measure of responses to stressors.

We conducted our experiment on free-living red grouse (*Lagopus lagopus scoticus*). This species displays supra-orbital red combs whose size is testosterone-dependent [19] and function in intra- and inter-sexual selection [20], [21]. A significant parasite of red grouse is the nematode *Trichostrongylus tenuis*, which has well known negative effects on this host: [22], [23]. In males, both testosterone and *T. tenuis* influence ornament expression, and interact: testosterone enhances ornamentation, impairs cellular immune responses [24] and increases susceptibility to *T. tenuis* [10], [25], while the parasites limit testosterone-dependent ornamentation [26]. Male red grouse with bigger combs are dominant, more aggressive and more successful at acquiring or maintaining larger territories [27]. In captivity, dominant males can suppress ornament expression in subordinates [27], [28]. In some territorial birds, subordinates show higher baseline levels of circulating corticosterone than dominant individuals, and losing fights can increase glucocorticoid production [29]. Therefore, there is substantial evidence for potential links between parasites, the endocrine basis of aggression and dominance (testosterone), stress (corticosterone), and ornamentation (comb area). We timed our experiment in autumn, when testosterone-dependent aggression and ornamentation plays a crucial role in recruitment, territory establishment and pairing for subsequent breeding [20], [30]. Autumn is also a time when parasite infection levels are at their highest [21], [22].

While there is considerable individual variation in behavioral and physiological responses to environmental perturbations [31], [32], the vast majority of studies has examined population- or treatment-level responses. While informative, they give little insight into individual differences in the ability to respond to stress and thus how natural or sexual selection may be operating. Thus, while we are interested in exploring treatment effects on HPA activity as measured in terms of corticosterone deposition in feathers grown during the course of the experiment, more importantly we focus on how individual variation in feather corticosterone may influence the impact of treatments on ornamentation and parasite susceptibility. We manipulated levels of testosterone (T) (by implantation; hormonal treatment or HTREAT) and parasites (through controlled infection; parasite treatment or PTREAT), and measured level of stress as evaluated by corticosterone (CORT) in feathers (Table 1). At the level of the treatments, we predicted (1) that increased testosterone levels would a) enhance ornamentation (increased comb area) and could b) increase parasite abundance after challenge, under the T-induced immunosuppression paradigm [5]. (2) Parasite challenges would reduce ornamentation and increase corticosterone levels. At the level of the individual, we predicted differences in CORT will explain a) *T. tenuis* abundance, given the immunosuppressive effects of CORT, and b) ornamentation, as less stressed males should be able or willing to increase comb area more than those experiencing higher stress levels.

**Table 1:** Overview of the timing of the experiment, procedures and sampling.

Event	Initial capture (C0)	First recapture (C1)	Second recapture (C2)
Date	25 Sept.±5 days	10 Oct.±5 days	27 Oct.±2 days
Procedure	Dosing with	Experiment start	Experiment end
	Anthelminthic (purging of *T. tenuis* worms)	-Testosterone treatment	
		-Parasite treatment	
Data sampling		*T. tenuis* parasites	*T. tenuis* parasites
		Testosterone	Testosterone
		Comb area	Comb area
Corticosterone assessment		primary feather	Re-grown primary feather collected for corticosterone analysis
		plucked	
		←Corticosterone deposited between C1 and C2→	

We show that administration of testosterone and parasites both impact ornament expression, but the response of an individual to both is largely explained by its exposure and physiological reaction to stressors as measured by corticosterone levels in feathers.

## Materials and Methods

### Experimental protocol

We conducted the experiment on two grouse moors (Edinglassie, northeast Scotland, Aberdeenshire, and Catterick, North Yorkshire, hereafter Moor 1 and Moor 2, respectively). In September 2006, we caught 40 adult male red grouse (20 per site), by night lighting and netting [22]. Upon first capture (C0; Table 1), each was fitted with a radio-collar (TW3-necklace radio-tags, Biotrack), given a 1 ml oral dose of a anthelminthic (Nilverm Gold, Schering-Plough Animal Health, Welwyn Garden City, UK) to purge it of *T. tenuis* nematodes [22], [26] and released at the capture site.

We started the experiment c. 15 days later (Table 1) allowing birds to clear the anthelminthic drug before parasite challenges. Upon this first recapture (C1), we randomly assigned each male to one of four treatments (five males / treatment / site):

1. Control implants (empty), no parasite challenge (T0P0);
2. Control implants, challenge with *T. tenuis* infective larvae (T0P+);
3. Testosterone implants, no parasite challenge (T+P0);
4. Testosterone implants, challenge with *T. tenuis* infective larvae (T+P+).

Males were implanted with two silastic tubes (20 mm long, 0.62 mm inner and 0.95 mm outer diameter) sealed with glue at both ends. T0 males were given two empty implants, and T+ males two implants filled with crystalline testosterone proprionate (Sigma Aldrich, UK) to elevate testosterone for 2–3 months [20]. Implants were inserted subcutaneously on the breast following local anesthesia. Challenged (P+) males received an oral dose of water containing c. 5000 *T. tenuis* infective larvae previously cultivated in the lab (see below), and P0 males were given water only (no challenge).

We recaptured males c. 17 days after hormone implants and parasite challenges (second recapture or C2) and ended the experiment (Table 1). At C1 and C2, we measured the maximum length and width of flattened comb with a ruler (nearest 1 mm; Table 1). We calculated comb area (length×width) as an index of ornament size [19]. We also took a blood sample from the brachial vein for T assays at C1 and C2. We immediately separated plasma by centrifugation (2 min at 7000 rpm) and froze the samples in liquid nitrogen within 5 min of collection. At C1, we plucked primary feather number one (innermost) on the right wing of each male. At C2, we plucked the re-grown primary for CORT analysis (see below). We thus sampled males to measure: (1) changes in plasma T concentration between C1 and C2, (2) *T. tenuis* abundance 17 d after challenge (at C2), (3) changes in ornamentation (comb area) between C1 and C2, (4) the amount of CORT deposited in the feather grown between C1 and C2. Sample sizes (number of males in each treatment group and site) are given in Table 2.

**Table 2:** Sample sizes for the data sampling according to treatment (4 groups), site (moor 1 or 2) and sampling time (C1 or C2).

Site	Moor 1				Moor 2				Total
Treatment	T0P0	T0P+	T+P0	T+P+	T0P0	T0P+	T+P0	T+P+	
Comb size at C1	5	5	5	5	5	5	5	5	40
Comb size at C2	5	5	5	5	5	5	5	3	38
Testosterone at C1	3	5	4	4	3	4	5	3	31
Testosterone at C2	3	5	2	3	3	2	4	2	24
Worms at C2	4	3	3	3	5	4	5	3	29
Feather CORT C1–C2	5	5	5	5	4	4	4	2	33

We held all the necessary UK Home Office licenses for conducting the procedures described in this work (PPL80/1437).

### Parasite abundance estimates and culture for challenges

*T. tenuis* is a significant parasite of red grouse. This gut nematode has a direct life style and no alternative hosts within the same habitat. Eggs laid by adult worms are voided onto the moor in cecal droppings, where they develop into infective larvae and are ingested by grouse when feeding on heather *Calluna vulgaris* [22]. We estimated *T. tenuis* parasite abundance using cecal egg counts at C1, and using direct worm counts from cecae collected from the birds (see also Materials and Methods S1), which were humanely killed (by dislocation of the neck) at the end of the experiment (C2). Sample size was unbalanced because we did not obtain cecal samples for parasite counts at C1 from all males. Cecal egg counts provide reliable estimates of worm burdens and were used to calculate worm abundance [33]. We used the remains of cecal samples collected upon first capture to cultivate *T. tenuis* parasite infective larvae for the subsequent challenges (see also Materials and Methods S1).

### Hormone assays

We measured plasma T concentration using a commercially available testosterone enzyme immunoassay (Elisa Kit EIA-1559 from DRG Diagnostics, Marburg, Germany), an assay which has been developed and validated for determining testosterone levels in small volume (20 µL) avian plasma samples [34] (see also Materials and Methods S1).We extracted CORT from feathers using a methanol-based extraction technique (complete details including validation of the methodology are presented in [18], [35] and see Materials and Methods S1). Data are expressed as pg CORT per mm, not per mg, of feather. The reason for doing so involves the nature of CORT deposition in feathers. CORT is deposited per unit time of feather growth which is approximated by length. Mass variation along the feather, or among parts of the feather, creates biases as concentrations become diluted as feather mass increases. When CORT per mm is used, one can track changes in the amount of hormone (including response to stressors) over time by measuring the proximal-distal variation [35]. Whole feathers were used in this study to measure CORT averaged over as long a period as possible, in this case the treatment period.

### Statistical analyses

We used SAS 8.01 SAS, 2001. Comb area, T and CORT concentrations were log-transformed and fitted to models using a normal error distribution (GLM or Mixed procedures; SAS, 2001). Counts of *T. tenuis* worms were fitted to models using a negative binomial error distribution (Genmod procedure; SAS, 2001). Parasite abundance estimates are given as geometric means±s.d. We examine the data with respect to hormone treatment (HTREAT; empty or T implants), parasite treatment (PTREAT; challenged or not challenged) and their possible interaction (HTREAT×PTREAT). We included “site” as a fixed effect in all models to control for possible site effects.

Comb area and T levels were measured prior to and after treatments, so we tested for treatment effects on changes over time using Generalized Linear Mixed Models (Mixed procedure in SAS). Initial models included time (before and after treatment), HTREAT, PTREAT and all interactions between these variables. Models included “individual” as a random effect, to account for repeated measures and variation within individuals. In contrast, CORT and parasites were measured only at the end of the experiment. We tested for treatment effects on these variables by including HTREAT, PTREAT and their interaction as explanatory variables.

We tested whether individual variation in feather CORT explained treatment effects on changes over time in comb area or parasite intensity at the end of the experiment using GLMs. Initial models included HTREAT, PTREAT, CORT, as well as all interactions. We subsequently removed non-significant terms, starting with interactions. Individual changes over time in comb area were calculated as the difference between final and initial values, corrected for initial values (residuals from a GLM of the difference on the initial value).

## Results

### Effect of treatments on *T. tenuis* abundance

At the time of implanting (C1), birds that had been dosed previously with anthelminthic (at C0) had no detectable *T. tenuis* worms (*n* = 5, 4, 7, and 10 for T0P0, T+P0, T0P+, T+P+ males, respectively). Thus, the initial anthelminthic treatment was effective at purging *T. tenuis*. At the end of the experiment (C2), variation in *T. tenuis* abundance was explained by parasite treatment (Genmod; PTREAT: *F*_1,25_ = 6.34; *p* = 0.011), but not by site (*F*_1,25_ = 2.66; *p* = 0.10), hormone treatment (HTREAT: *F*_1,25_ = 0.01; *p* = 0.95) or the interaction PTREAT×HTREAT (*F*_1,25_ = 0.00; *p* = 0.97). T0P0 males had, on average (geometric mean±s.d.), 16±33 worms (*n* = 9), T0P+ males 205±383 worms (*n* = 9), T+P0 males 42±90 worms (*n* = 6) and T+P+ males 88±89 worms (*n* = 6). Thus *T. tenuis* abundance was higher in parasite challenged than non-challenged males, irrespective of T treatment.

### Effect of treatments on testosterone concentration and comb area

**Figure 1:**
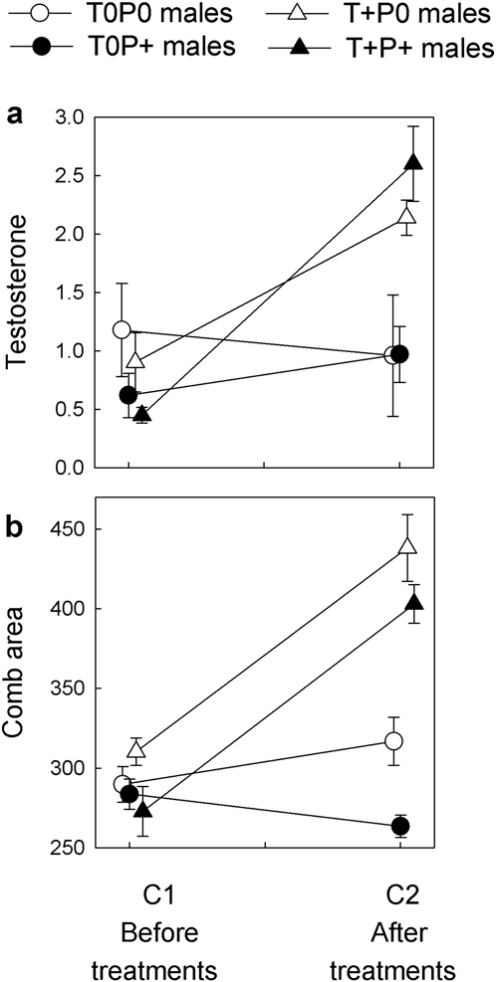
Effects of testosterone and parasite treatments on mean±s.e. (a) plasma concentration of testosterone (ng /ml) and (b) comb area (mm^2^) of red grouse. T0: control implanted; T+: testosterone implanted; P0: purged of worms; P+: challenged with *T. tenuis* infective larvae.

Temporal changes in testosterone concentration (T), between implanting (C1) and re-capture (C2), differed significantly between treatment groups (Table 3; Fig. 1a). T concentration did not differ between sites (Table 3), and increased significantly more in T+ males than in T0 males (significant Time×HTREAT interaction; Table 3 Fig. 1a). Parasite treatment had no significant effects on changes in T (Table 3: non-significant Time×PTREAT and Time×PTREAT×HTREAT interactions; Fig. 1a).

**Table 3:** Generalised Linear Mixed Models testing for treatment effects on changes over time in testosterone concentration and ornamentation (comb area).

Dependent variables:	Testosterone*			Comb area*		
	df	F	P	df	F	P
Site	1,13	2.27	0.16	1,33	0.24	0.63
Time†	1,13	72.77	<0.001	1,33	61.97	<0.001
HTREAT‡	1,13	17.87	<0.001	1,33	24.74	<0.001
PTREAT∥	1,13	0.09	0.76	1,33	10.09	0.0032
Time×HTREAT	1,13	29.67	<0.001	1,33	75.89	<0.001
Time×PTREAT	1,13	0.89	0.36	1,33	1.57	0.22
HTREAT×PTREAT	1,13	0.17	0.69	1,33	0.14	0.71
Time×HTREAT×PTREAT	1,13	2.83	0.12	1,33	6.23	0.0177

* The dependent variables were log-transformed. Sequential model outputs are given.

† Time: C1 (first recapture, when treatments were given) vs C2 (second recapture, 17 days after treatments). See Table 1.

‡ HTREAT: Hormone treatments (T0: control implanted vs T+: testosterone implanted).

∥ PTREAT: Parasite treatments (P0: purged of worms; P+: challenged with *T. tenuis* infective larvae).

Changes in comb area over time differed significantly between treatment groups (Table 3; Fig. 1b). Comb area did not differ between sites (Table 3) and increased more in T+ males than in T0 males between C1 and C2 (Table 3: significant Time×HTREAT interaction; Fig. 1b). Parasite manipulations affected changes in comb area, but depending on hormone treatment (Table 3: significant Time×PTREAT×HTREAT interaction; Fig. 1b). To clarify this interaction, we analyzed PTREAT effects in T0 and T+ males separately. In T+ males, parasite treatments had no significant effect on changes in comb area (non-significant Time×PTREAT interaction: *F*_1,14_ = 0.63; *p* = 0.44). In T0 males, however, parasite treatment affected changes in comb area (significant Time×PTREAT interaction: *F*_1,19_ = 12.25; *p* = 0.0024); comb area decreased more in challenged (T0P+) males than in non-challenged (T0P0) males (Fig. 1b).

### Effects of treatments on corticosterone

**Figure 2:**
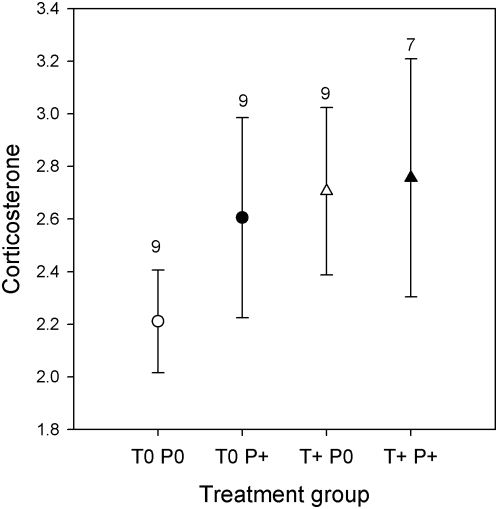
Mean±s.e. levels of corticosterone (pg / mm) in feathers of red grouse according to hormone and parasite treatments. T0: control implanted; T+: testosterone implanted; P0: purged of worms; P+: challenged with *T. tenuis* infective larvae. Numbers above bars are sample sizes.

CORT levels deposited in feathers grown between C1 and C2 did not differ between sites (*F*_1,30_ = 0.19; *p* = 0.67) or treatments (HTREAT: *F*_1,30_ = 1.09; *p* = 0.31; PTREAT: *F*_1,30_ = 0.31; *p* = 0.59; HTREAT×PTREAT: *F*_1,30_ = 0.04; *p* = 0.84). Thus, although the T implants increased testosterone concentration and the parasite challenges increased *T. tenuis* abundance, these treatments did not generate differences in CORT, although there was considerable individual variation within treatment groups (Fig. 2).

### Corticosterone and treatment effects on *T. tenuis* parasites

**Figure 3:**
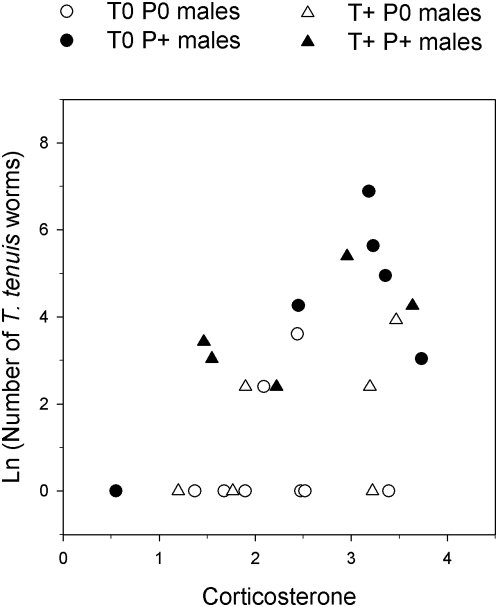
Relationship between corticosterone (pg/mm) in feathers and final number of *T. tenuis* worms in red grouse by treatment. T0: control implanted; T+: testosterone implanted; P0: purged of worms; P+: challenged with *T. tenuis* infective larvae.

We tested whether individual variation in CORT deposited between C1 and C2 explained treatment effects on parasite abundance at the end of the experiment. The initial models included site, and all single factors and interactions between HTREAT, PTREAT and CORT. Variation in final parasite intensity was not explained by site, HTREAT, nor interactions between HTREAT, PTREAT and CORT (all *p*>0.13). The final model only retained PTREAT and CORT as significant explanatory variables: final parasite intensity was higher in challenged than in non-challenged males (PTREAT: *F*_1,22_ = 4.55; *p* = 0.033) and was significantly positively related to CORT (*F*_1,22_ = 9.38; *p* = 0.002; Fig. 3).

### Corticosterone and treatment effects on ornamentation

**Figure 4:**
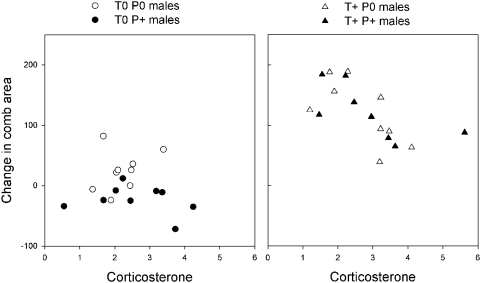
Relationship between corticosterone (pg/mm) in feathers and changes in comb area (mm^2^) of male red grouse between C1 and C2, according to treatment. a) T0: control implanted males; b) T+: testosterone implanted males. Parasite treatments: P0: purged of worms; P+: challenged with *T. tenuis* infective larvae.

We tested whether individual variation in CORT explained treatment effects on male ornamentation. Changes in comb area between C1 and C2 were explained by HTREAT (*F*_1,29_ = 23.01; *p*<0.001), PTREAT (*F*_1,29_ = 4.84; *p* = 0.036), the PTREAT×HTREAT interaction (*F*_1,29_ = 4.45; *p* = 0.044), CORT (*F*_1,29_ = 5.09; *p*<0.001), and by the interaction CORT×HTREAT (*F*_1,29_ = 5.96; *p* = 0.021; model: R^2^ = 0.82; Fig. 4 a,b). Site, and the interactions CORT×PTREAT and CORT×PTREAT×HTREAT were not significant and not retained in the final model (all *p*>0.21). To clarify the interactions, we analysed changes in comb area by hormone treatment (HTREAT).

In T0 males, changes in comb area were explained by the parasite treatment (PTREAT; *F*_1,14_ = 12.19; *p* = 0.003), but not by CORT (*F*_1,14_ = 0.02; *p* = 0.89) or the interaction CORT×PTREAT (*F*_1,14_ = 0.55; *p* = 0.47). Comb area decreased more in challenged males (T0P+) than in non-challenged males (T0P0; Fig. 4a).

In T+ males, changes in comb area were only explained by CORT (*F*_1,13_ = 8.81; *p* = 0.011), and not parasite treatment (PTREAT: *F*_1,13_ = 0.43; *p* = 0.523; CORT×PTREAT: *F*_1,13_ = 0.43; *p* = 0.52). Higher CORT levels were associated with a reduced increase in comb area (Fig. 4b), with variation in this hormone alone explaining 41% of the effect of T implants on changes in ornamentation.

## Discussion

### Treatment level effects

Our hormonal treatments successfully created differences in testosterone levels between groups, about two times higher in T+ than in T0 males, but that were still within the natural range of the species [19]. Parasite challenges also increased parasite abundance (higher in P+ males compared to P0 males); however, we found no short-term effect of elevated T on parasite intensity after a standardized parasite challenge, as predicted under a under the T-induced immunosuppression paradigm. In previous experiments, elevated T increased parasite intensity but with a time lag of 4–12 months [10], [25], [36], probably because some larvae arrest their development [37], [38], and so the effect of T on parasites might not be fully appreciated less than a month after challenge. Alternatively, T might not have a direct, immunosuppressive effect on parasite susceptibility [10], and might increase parasite susceptibility only when elevated T also increases CORT levels. This would be consistent with our observation that elevated T did not increase CORT at the treatment group level (Fig. 2), although individual CORT variation explained parasite abundance after challenges (Fig. 3).

As expected, elevated T enhanced ornamentation, while parasite challenges reduced ornamentation, but only in control-implanted males (Fig. 1b). Parasite challenges might not have reduced ornamentation in T+ males because T implants forced males to maintain high testosterone levels, irrespective of parasites (see Fig. 1a).

### Individual variation and stress as a relevant context

While the administration of testosterone and parasites both impacted ornament expression, the response of an individual to both was largely explained by its exposure and physiological reaction to stressors as measured by corticosterone levels in feathers. CORT and parasite treatment explained how many parasites males had at the end of the experiment, consistent with an effect of stress hormones on parasite susceptibility. CORT also explained by how much males increased ornamentation when implanted with testosterone. This shows that the interplay between the individual and its environment, evaluated through an integrated measure of response to stressors, impacts both health (at least parasites) and ornamentation.

Many physiological processes are characterized by a high degree of variability among individuals, something that eco-physiologists are struggling to come to grips with [32]. What our study has done is to provide a context, the production of corticosterone, which we propose is indicative of responses to environmental perturbations, to better interpret why one individual is more likely to be parasitized and/or otherwise struggle to develop essential, social signals. Such information is crucial if we are to understand how or where selection may be operating.

Our results help explain the apparent discrepancies in tests of the Immunocompetence Handicap Hypothesis [7]. Perez-Rodriguez et al. (2006), in their investigation of androgens and energy stores in captive red-legged partridges (*Alectoris rufa*), proposed that variation in the relationship between immunocompetence and testosterone was within the realm of the IHH. When they experimentally restricted food intake and body condition declined, circulating androgens also decreased. They proposed that there may be a threshold between condition and testosterone production such that the honest signaling of testosterone-dependent ornaments (via immunocompetence) may only be evident in individuals “free of the constraints imposed by nutritional status” [39]. Our findings here suggest that instead of body condition per se being causal, food restriction could be viewed as food stress; concomitant with the fall in androgens was an increase in corticosterone [39]. The fact that CORT explained a sizeable portion of the variation in T-dependent ornament size in the grouse of the T+ but not the T0 group, also suggests a potential threshold effect and further demonstrates the importance of environmental context [40]. Experiments on genetic lines of zebra finches (*Taeniopygia guttata*) artificially selected for high and low levels of circulating CORT failed to demonstrate any effect on ornament expression or choice of mate [41], [42]. However, such results are not unexpected if the honesty of signals involve a threshold, or at least be more indicative of honesty under challenging circumstances when true individual quality will have its most significant impact on fitness. While the differences in the genotypes of zebra finches had the potential to respond to the environment in a different way, they may not have had the opportunity to do so and thus signal differences in quality.

### The role of stress

How well an animal copes with the many, often concurrent, challenges in the environment may be one of the more meaningful, if not the best, overall measure of its “quality” and fitness potential. Despite the fact that individual “quality” is the cornerstone of honest signaling, most studies of sexually selected ornamentation have only alluded to stress, or the ability to respond to it, in a general fashion [43]. Glucocorticoids are only one of many physiological mediators of the response to environmental perturbations, but they play crucial roles [16], [44]. Because physical and psychological stressors induce a similar physiological response, the production of glucocorticoids may provide a common currency with which to evaluate and compare individual performance in any part of the life history cycle. The CORT we are measuring in feathers includes more physiological processes that what can be attributed to the response to stress alone. However, the spike in circulating corticosterone in response to a life-threatening situation has a disproportionately influence on feather levels [18].

There is intuitive appeal to the logic that ornamentation is ultimately a product of the cumulative physiological response to environmental challenges. From a behavioral perspective, it may matter little to a female who is about to choose a mate, or a male who is about to battle a rival, what combination of all possible stressors the subject has experienced. What matters, is that the individual has either been able to avoid them (e.g., by virtue of a superior territory) or coped well with them (e.g., better genes, better condition). This helps to explain why the same ornament can be used as an honest signal by a species under different selection regimes, e.g., a wide geographic distribution encompassing various environmental challenges. A truly reliable indicator of quality should have such flexibility. A broader perspective on the costs of adaptation to a changing environment, and a focus on the individual, is a step toward a more comprehensive and hopefully realistic concept of honesty in signaling.

## Supporting Information

Materials and Methods S1 (0.07 MB DOC)
