# Supplementary material for: Physiological Stress Mediates the Honesty of Social Signals
Source: PLoS One. 2009 Mar 25;4(3):e4983. doi: 10.1371/journal.pone.0004983 (PMC2656621; doi:10.1371/journal.pone.0004983)
Supplement: Materials and Methods S1 — (0.07 MB DOC) [file pone.0004983.s001.doc]

**Supporting information**

**Additional Materials and Methods**

**Testosterone assays.** We measured plasma T concentration using a commercially available testosterone enzyme immunoassay (Elisa Kit EIA-1559from DRG Diagnostics, Marburg, Germany), an assay which has been developed and validated for determining testosterone levels in small volume (20 μL) avian plasma samples [1]. Coefficient of variation intra- and inter-assays were 3.59 and 7.14, respectively, and the detection limit 0.2 ng/ml. Replicate sample analyses showed that the assays were repeatable (*F*_23,59_ = 15.99, *p* < 0.001; *r* = 0.88). Testosterone assays were performed at IREC, Spain.

**Corticosterone assays.** We extracted corticosterone (CORT) from feathers using a methanol-based extraction technique (complete details including validation of the methodology are presented in [2]). The length of the feather was measured, the calamus was removed and discarded and then the sample was minced into pieces <5 mm^2^ with scissors. We then added 10 mL of methanol (HPLC grade, VWR International, Mississauga, Ontario, Canada) and placed the samples in a sonicating water bath at room temperature for 30 min, followed by incubation at 50 °C overnight in a shaking water bath. The methanol was then separated from feather material by vacuum filtration, using a plug of synthetic polyester fibre in the filtration funnel. The feather remnants, original sample vial and filtration material were washed twice with approximately 2.5 mL of additional methanol; the washes were added to the original methanol extract. The methanol extract was placed in a 50 °C water bath and subsequently evaporated in a fume hood. Evaporation of the samples was completed within a few hours and the extract residues were reconstituted in a small volume of the phosphate buffer system (PBS; 0.05M, pH 7.6) used in the CORT radioimmunoassay [3]. The filtration step was generally found to be sufficient to remove feather particulates but further particulate material could be removed, if needed, by centrifugation of the PBS-reconstituted samples. Reconstituted samples were frozen at –20 °C until analyzed for CORT by RIA [3]. We assessed the efficiency of the methanol extraction by including feather samples spiked with a small amount (approximately 4000 DPM) of ^3^H-corticosterone in each extraction. More than 90% of the radioactivity was recoverable in the reconstituted samples. Data values are expressed as pg CORT per mm of feather, which gives a valid estimate of CORT per unit time of feather growth [2,4]. CORT assays were performed at the University of Saskatchewan, Canada.

**Parasite abundance estimates and culture for challenges.** The intensity of parasite eggs per gram of caecal faeces was estimated using a modified McMaster egg counting technique. Approximately 0.2 g (range 0.19–0.21 g) of well-mixed faecal material was put into a shaker tube with approximately 10 glass balls and 5ml of saturated NaCl solution. The tube was shaken until the faecal matter was suspended. Using a Pasteur pipette, a sample of the faecal suspension was extracted and carefully run into one chamber of a McMaster counting slide. The tube was shaken again and another sample extracted and run into the second section of the chamber. The saline suspension was left to settle for 2–3 min, allowing the eggs to float to the top of each chamber. Eggs were then counted beneath a marked grid on each chamber using a compound microscope with 100 x magnification. The number of eggs per gram of faeces was calculated by multiplying the total number of eggs counted under both grids by the total volume of faecal suspension contained in both chambers and then dividing this by the quantity of faeces used in the suspension. Caecal egg counts provide reliable estimates of the number of *T. tenuis* worms per grouse [5].

Direct worm counts were carried out on one caecum from each grouse. Each caecum was opened lengthways and all the gut material flushed with water over 150 mm gauze to collect the worms. The contents of the sieve were washed into 300 ml of water, mixed thoroughly and adult worms counted with the aid of a binocular dissecting microscope with 25 x magnification. All worms in the sample were counted and this number doubled to obtain the total number of worms per bird, as worm numbers do not significantly differ between the two caeca.

We used the remains of cecal samples collected upon first capture to cultivate *T. tenuis* parasite infective larvae for the subsequent challenges. Cecal samples were washed thoroughly over a 125 µm sieve (Endecotts Ltd., Moreden, London) to remove coarse, fibrous material. The residue containing eggs was collected with a 25 µm sieve using distilled water, placed in petri dishes and incubated at 20 °C. These cultures were mixed and watered daily to maintain optimal moisture conditions for the hatching of *T. tenuis* eggs [6]. After two weeks, the cecal contents were filtered and the larvae extracted and concentrated using a modified Baerman apparatus. Infective larvae were stored in distilled water for up to three days in a refrigerator at 4 °C. On the day prior to challenges, the solution containing the infective larvae was mixed and larvae concentration was measured by counting live larvae in sub samples. Individual doses containing c. 5000 infective larvae were then extracted and stored refrigerated until given orally to males. Further details on the cultivating, storing and counting *T. tenuis* larvae are given elsewhere [5-7].

**References**

1. Washburn BE, Millspaugh JJ, Morris DL, Schulz JH, Faaborg J (2007) Using a commercially available enzyme immunoassay to quantify testosterone in avian plasma. Condor 109: 181-186.

2. Bortolotti GR, Marchant TA, Blas J, German T (2008) Corticosterone in feathers is a long-term, integrated measure of avian stress physiology. Functional Ecology 22: 494-500.

3. Blas J, Baos R, Bortolotti GR, Marchant T, Hiraldo F (2005) A multi-tier approach to identifying environmental stress in altricial nestling birds. Functional Ecology 19: 315-322.

4. Bortolotti GR, Marchant T, Blas J, Cabezas S (2009) Tracking stress: localization, deposition and stability of corticosterone in feathers. Journal of Experimental Biology in press.

5. Seivwright LJ (2004) Pattern of *Trichostrongylus tenuis* infection in individual red grouse *Lagopus lagopus scoticus*. Stirling: University of Stirling.

6. Shaw JL (1988) Arrested development of *Trichostrongylus tenuis* as 3rd stage larvae in red grouse. Research in Veterinary Science 45: 256-258.

7. Moss R, Trenholm IB, Watson A, Parr R (1990) Parasitism, predation and survival of hen red grouse *Lagopus lagopus scoticus* in spring. Journal of Animal Ecology 59: 631-642.
